# Impact of asynchronous emergence of two lethal pathogens on amphibian assemblages

**DOI:** 10.1038/srep43260

**Published:** 2017-02-27

**Authors:** Gonçalo M. Rosa, Joana Sabino-Pinto, Telma G. Laurentino, An Martel, Frank Pasmans, Rui Rebelo, Richard A. Griffiths, Anke C. Stöhr, Rachel E. Marschang, Stephen J. Price, Trenton W. J. Garner, Jaime Bosch

**Affiliations:** 1 Durrell Institute of Conservation and Ecology, School of Anthropology and Conservation, University of Kent, Canterbury, Kent, CT2 7NR, UK; 2 Institute of Zoology, Zoological Society of London, Regent’s Park, NW1 4RY, London, UK; 3 Centre for Ecology, Evolution and Environmental Changes (CE3C), Faculdade de Ciências da Universidade de Lisboa, Campo Grande, 1749-016 Lisboa, Portugal; 4 Department of Biology, University of Nevada, Reno, NV 89557, USA; 5 Technische Universität Braunschweig, Division of Evolutionary Biology, Zoological Institute, Mendelssohnstr. 4, 38106 Braunschweig, Germany; 6 Computational Biology and Population Genomics Group, Centre for Ecology, Evolution and Environmental Changes (CE3C), Faculdade de Ciências da Universidade de Lisboa, Campo Grande, 1749-016 Lisboa, Portugal; 7 Zoological Institute, University of Basel, Vesalgasse 1, Basel, Switzerland; 8 Department of Pathology, Bacteriology and Avian Diseases, Faculty of Veterinary Medicine, Ghent University, Ghent, Belgium; 9 Fachgebiet für Umwelt- und Tierhygiene, Universität Hohenheim, Stuttgart, Germany; 10 Laboklin GmbH & Co. KG, Laboratory for Clinical Diagnostics, Bad Kissingen, Germany; 11 UCL Genetics Institute, Gower Street, London, WC1E 6BT, UK; 12 Museo Nacional de Ciencias Naturales, CSIC, José Gutiérrez Abascal 2, 28006 Madrid, Spain

## Abstract

Emerging diseases have been increasingly associated with population declines, with co-infections exhibiting many types of interactions. The chytrid fungus (*Batrachochytrium dendrobatidis*) and ranaviruses have extraordinarily broad host ranges, however co-infection dynamics have been largely overlooked. We investigated the pattern of co-occurrence of these two pathogens in an amphibian assemblage in Serra da Estrela (Portugal). The detection of chytridiomycosis in Portugal was linked to population declines of midwife-toads (*Alytes obstetricans*). The asynchronous and subsequent emergence of a second pathogen - ranavirus - caused episodes of lethal ranavirosis. Chytrid effects were limited to high altitudes and a single host, while ranavirus was highly pathogenic across multiple hosts, life-stages and altitudinal range. This new strain (Portuguese newt and toad ranavirus – member of the CMTV clade) caused annual mass die-offs, similar in host range and rapidity of declines to other locations in Iberia affected by CMTV-like ranaviruses. However, ranavirus was not always associated with disease, mortality and declines, contrasting with previous reports on Iberian CMTV-like ranavirosis. We found little evidence that pre-existing chytrid emergence was associated with ranavirus and the emergence of ranavirosis. Despite the lack of cumulative or amplified effects, ranavirus drove declines of host assemblages and changed host community composition and structure, posing a grave threat to all amphibian populations.

Geographic ranges of pathogens are dynamic, generating novel interactions with potential host species and their existing pathogens^1^,^2^,^3^. The outcomes of these processes for host populations are difficult to forecast since the effects on the likelihood of disease are unpredictable^4^,^5^,^6^. Most of our understanding of the nature of multi-pathogen interactions in multi-host communities is derived from experimental evidence, or inferred from cross-sectional studies of infections within a host community^7^,^8^. Longitudinal studies of co-infection dynamics in wild animal host communities are exceptional (e.g. refs 5 and 9) and even then, the initiation of a two-pathogen interaction is almost never captured by field studies. Data on the latter would be of great interest, as experimental evidence shows that the point at which the interaction is initiated can significantly affect disease outcomes and demography of host populations^10^,^11^.

Biodiversity is increasingly threatened by infectious disease emergence associated with rapid population declines^12^,^13^,^14^,^15^. Most of these are multi-host pathogens, none more so than the amphibian-associated chytridomycete fungi and ranaviruses. These pathogens have an extraordinarily broad host range, infecting dozens of amphibian species and, in the case of the ranaviruses, reptile and fish hosts as well^16^,^17^. Both are responsible for amphibian mass mortality events across the globe, many of which involve multiple host species^12^,^13^,^18^,^19^. However, not all cases of ranavirosis and chytridiomycosis affect a broad host range^20^,^21^,^22^,^23^,^24^,^25^ and many host populations sustain recurring mortality events without any evidence of demographic decline, or even carry infections without overt signs of disease^21^,^23^,^26^. Variation of pathogenic effects and virulence have been attributed to host and pathogen genotypes^27^, environmental factors^28^ and variation of host immunity (e.g. ref. 29), but what is increasingly recognized is that these two pathogen groups commonly overlap in range and co-infect amphibian hosts^30^,^31^,^32^,^33^. These cross-sectional studies raise the question as to whether impacts on host populations and communities are caused by one or the other pathogen group, or both^34^,^35^. Notwithstanding, the majority of studies focus exclusively on one or the other pathogen group and, as a result, co-infection dynamics are being understudied^33^,^36^.

Iberia is Europe’s worst affected region given the serious outcomes that have arisen from the lethal forms of both diseases^13^,^23^,^37^. Chytridiomycosis attributable to infection with *Batrachochytrium dendrobatidis (Bd*) has been assessed to some degree and shown to be a risk to Iberian amphibian host species, but only in the context of single pathogen effects^23^,^24^,^28^,^37^,^38^,^39^,^40^,^41^,^42^. Recently, cases of lethal ranavirosis that have emerged in the region are responsible for amphibian host community collapses^13^. Again, these cases are considered only from the single pathogen perspective, but do suggest that lethal ranavirosis is an emerging disease in Iberia and show that this pathogen affects the same hosts as lethal chytridiomycosis in the region.

We previously reported lethal chytridiomycosis responsible for mass mortality of common midwife toads (*Alytes obstetricans*) inhabiting Serra da Estrela, Portugal^24^. Since then we have been monitoring amphibian communities in the area and here report the emergence of lethal ranavirosis in the same region. This allowed us the novel opportunity to ascertain the distribution of the two pathogens in amphibian assemblages in Serra da Estrela. In turn, by closely monitoring index sites for emergent ranavirosis, we perform both quantitative and qualitative assessments of the impact of infectious disease on host communities in the presence of one (*Bd*) or both pathogens. Here we report the results of these surveys, spanning six years of surveillance, where we documented sequential emergence of lethal chytridiomycosis and ranavirosis.

## Results

Up until the summer of 2011, all the mortality events at Serra da Estrela Natural Park were only associated with the presence of *Bd. Alytes obstetricans* metamorphs dying at high elevation sites (above 1200 m) were confirmed to be infected with *Bd* and often dying as a result^24^. Although *Bd* was present at both Sazes and Folgosinho, we did not detect mortality clearly attributable to chytridiomycosis at these locations during this study. Only 5.7% (3/53) of dead *Lissotriton boscai* and just 4.4% (4/92) of all newts, live or dead, tested positive for *Bd* at Folgosinho across all years. Prevalence of infection of overwintering larvae of *Alytes* never exceeded 33.3%. No *Salamandra* tested positive for *Bd* and only two *Triturus marmoratus* tested positive at Folgosinho (Table S3).

**Figure 1:**
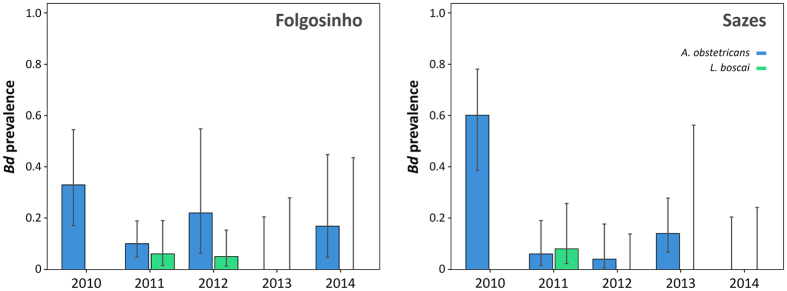
Prevalence of *Batrachochytridium dendrobatidis* in common midwife toad (*Alytes obstetricans*) and Bosca’s newts (*Lissotriton boscai*) at two sites in Serra da Estrela (Portugal). No data available for *L. boscai* in 2010. Prevalence includes 95% confidence intervals (CIs).

Prevalence of infection with *Bd* varied between species (*L. boscai* and *A. obstetricans*) at Folgosinho and Sazes (*F* = 5.91, *df* = 1, 15, *P* = 0.0334), but not over time (*F* = 4.32, *df* = 1, 15, *P* = 0.0620) nor across the two study sites (*F* = 1.35, *df* = 1, 15, *P* = 0.2698; Fig. 1, Table S3). The presence/emergence of ranavirus was not a predictor of *Bd* variance (*F* = 0.23, *df* = 1, 15, *P* = 0.6449).

**Figure 2:**
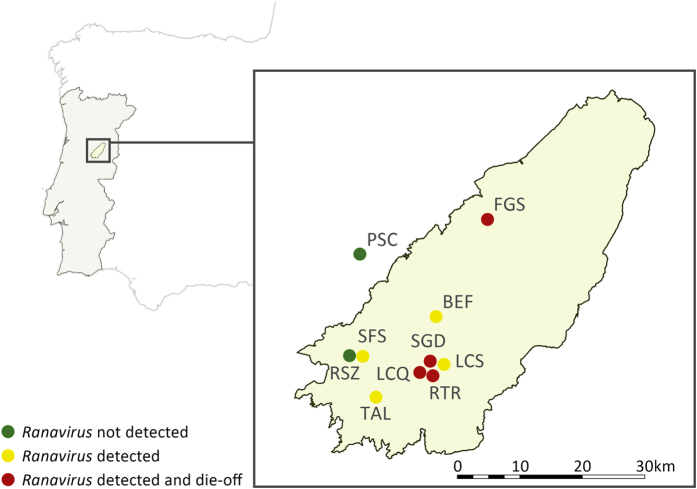
Site locations for ranavirus infection and die-offs in amphibian assemblages of Serra da Estrela Natural Park between 2011–2014. Abbreviations key: BEF, Barragem da Erva da Fome; LCQ, Lagoa do Covão das Quelhas; LCS, Lagoa dos Cântaros; PSC, Pedreira de Santa Comba de Seia; RTR, Represa da Torre; RSZ, Repreza de Sazes; SGD, Salgadeiras; FGS, Tanque de Folgosinho; TAL, Tanque do Alvoco; SFS, Tanque dos Serviços Florestais de Sazes. Points on the map were generated using QGis 2.0 (Quantum GIS Development Team, 2013) and edited in Adobe PhotoShop CS6 (Adobe, 2012).

**Figure 3:**
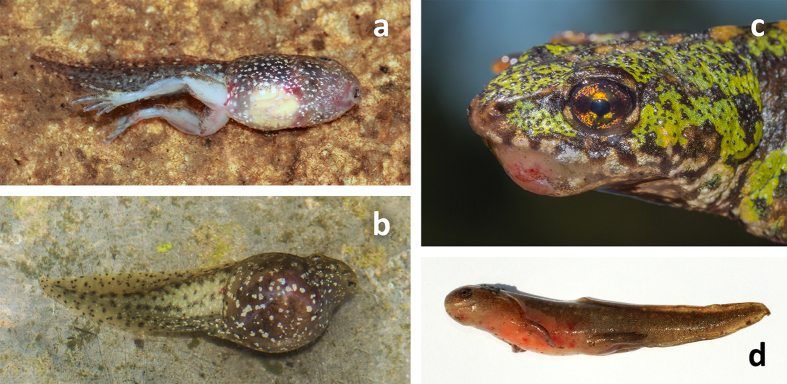
Ranavirosis in several species of amphibians in Serra da Estrela (Portugal). (**a,b**) Dead larvae of *Alytes obstetricans* presenting internal hemorrhages and bloating; (**c**) Live adult *Triturus marmoratus* with superficial and ulcerating skin lesions; (**d**) *Lissotriton boscai* larva with skin haemorrhages and ulcerations. Photos by G. M. Rosa.

**Figure 4:**
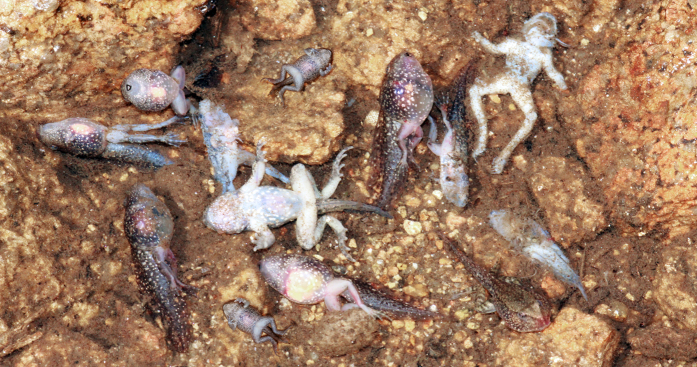
Mass mortality episode in Serra da Estrela, Portugal (summer 2013). Dead *Alytes obstetricans* and *Bufo spinosus* tested positive for both *Bd* and *Ranavirus*. Photo by G. M. Rosa.

Ranavirus infections were detected throughout Serra da Estrela Natural Park, despite small sample sizes for some of the sites (Fig. 2; Table S3). Prior to the summer of 2011, we never encountered dead amphibians that tested positive for ranavirus through molecular analyses or that presented overt signs of ranavirosis. We first detected infection with ranavirus in August of 2011, when two live adult *T. marmoratus* sampled at Folgosinho and several species sampled at Represa da Torre tested positive by PCR (Table S3). At that time mortality was observed in recently metamorphosed individuals of *Alytes obstetricans* and *Bufo spinosus*. When we returned to Folgosinho in November of that same year, 92.3% of the Bosca’s newts found at the site were dead and exhibited overt signs of ranavirosis. Sick/moribund and dead animals exhibited skin haemorrhages on their ventral body surfaces, ulcerations and, in a few cases, limb necrosis - all gross signs typical of lethal ranavirosis (Fig. 3^43^). Tissues were necrotic, with cells in cytolysis with only nuclei remaining, severely limiting the examination. Nevertheless, we could see that the skins of necropsied individuals presented greyish foci and focal erythema associated with enlarged, mottled pale brown and friable livers. Mortality of *L. boscai* was recorded across all life stages and ages making use of the aquatic environment (Fig. S1). Ranavirus prevalence as determined using PCR was extremely high (96% for *L. boscai*, and 90% across all species; Table 1, S1). The same pattern of mass mortality, involving multiple amphibian species, was repeated annually across all four seasons at Folgosinho. Each year we encountered numerous dead and/or dying adult and larval caudates (*L. boscai, T. marmoratus* and *S. salamandra*) and *A. obstetricans*. In addition to Represa da Torre and Folgosinho, we observed and confirmed lethal ranavirosis at two other locations in the park (Fig. 2, Table S3). Here, *A. obstetricans* of all life history stages were found dead or dying and exhibiting clinical signs of ranavirosis, and ranavirus infection was confirmed with molecular diagnostics. However, many of these animals also tested positive for infection with *Bd* (Fig. 4; Table S3). Co-infections were detected in *Hyla molleri, B. spinosus* and *L. boscai*. Lethal chytridiomycosis had previously caused mass mortality of *A. obstetricans* at all three of these higher elevation locations, but lethal disease had only been reported to affect recently metamorphosed juveniles^24^. By comparison, we recorded only 4/233 ranavirus-infected individuals at Sazes over the same time span (Table S3) and never observed any overt signs of disease.

**Figure 5:**
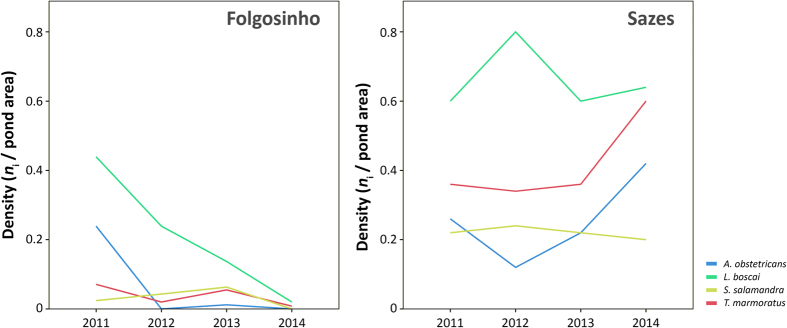
Population trends of two amphibian communities in Serra da Estrela (Portugal) over four years (spring counts). At Folgosinho density per species after 2011 significantly decreases coincidentally with yearly outbreaks of ranaviruses, while density fluctuations of an assemblage at Sazes where outbreaks have not been recorded show no such pattern. Life history stage varied with species but was consistent for each site across years: *Alytes obstetricans* (overwintering larvae), *Lissotriton boscai* (adults), *Triturus marmoratus* (adults) and *Salamandra salamandra* (larvae).

We found a highly significant effect of time on abundance for most of the species at Folgosinho, where those experienced a sharp decline coinciding with the first outbreaks of ranavirosis (Fig. 5; Table 1). As an example, the adult population of *L. boscai* declined by 45.5% between 2011 and 2012, and 68.8% between 2011 and 2013 (Fig. 5). In the spring of 2014 the Folgosinho tank was emptied, dramatically disturbing the amphibian community one month before our survey, thus compromising interpretation of population trends for 2013–2014. Nevertheless, abundance of three amphibian species experiencing lethal ranavirosis at this location declined by a minimum of 70%, and almost 100% for *L. boscai* and *A. obstetricans* when compared to 2011, before the ranavirosis outbreak (Fig. 5). Despite evidence of infection in *S. salamandra* (prevalence 5.3% across all years, Table S3) and our failure to detect high numbers of larvae of this species (Fig. 5), the density of *S. salamandra* larvae did not change significantly from 2011–2013. At Sazes, species trends fluctuated, but never exhibited the clear pattern of decline observed at Folgosinho (Fig. 5; Table 1).

**Figure 6:**
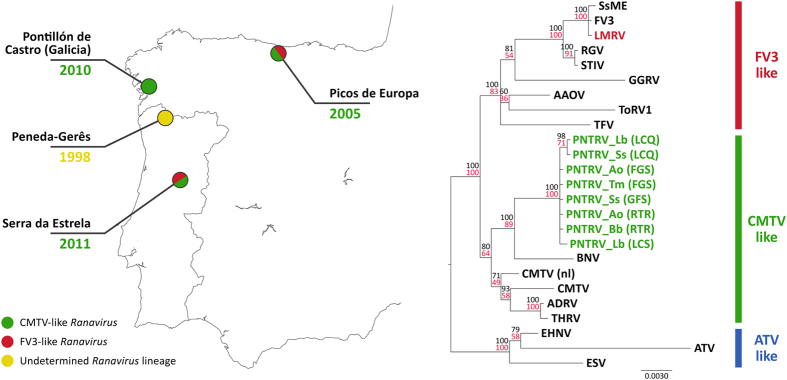
*Ranavirus* phylogeography of the Iberian Peninsula. Portuguese newt and toad ranavirus (PNTRV) relationships to other known Iberian ranaviruses of wild herpetofauna. Year of first observed outbreaks of CMTV-like ranaviruses (green), FV3-like ranaviruses (red) and an undetermined lineage (yellow). PNTRV infecting amphibians is embedded in the CMTV-like clade, while LMRV has been found in Serra da Estrela population of *Iberolacerta monticola* and is part of the FV3-like group. Phylogeny constructed from concatenated alignments of six partial genes (see main text). The final concatenated alignment was 2015 bp in length. Node support values were annotated on the best maximum-likelihood tree and were calculated using maximum likelihood (100 bootstraps, black) and Bayesian inference (posterior probabilities as percentage, red). Scale of branch lengths is in nucleotide substitutions per site. Abbreviations key: FGS, Tanque de Folgosinho; LCS, Lagoa dos Cântaros; RTR, Represa da Torre; LCQ, Lagoa do Covão das Quelhas. Points on the map were generated using QGis 2.0 (Quantum GIS Development Team, 2013) and edited on Adobe PhotoShop CS6 (Adobe, 2012).

Virus sequences were predominantly 100% identical to each other across all sequenced genes, and consistent with the recently identified Portuguese newt and toad ranavirus (PNTRV)^45^. Node support was high across our tree for clades involving viruses from this study. PNTRV clustered unambiguously in the “CMTV-like” group, the sister taxa to Bosca’s newt virus (BNV) isolated from newts that died from ranavirosis in Galicia, Spain (Fig. 6).

## Discussion

Common midwife toads (*A. obstetricans*) across Serra da Estrela Natural Park experienced significant population loss prior to 2009, when lethal chytridiomycosis was first detected affecting the species in the park^24^. *Batrachochytrium dendrobatidis* was already widespread in the area and declines due to chytridiomycosis were proposed as the cause of population losses^24^. Ranaviruses were unlikely to have played a role in local extirpation of *Alytes* before 2011, as carcasses subjected to post mortem examination in 2009 and 2010 and diagnosed with chytridiomycosis did not exhibit signs of ranavirosis^24^. Instead we first detected the presence of a CMTV-like ranavirus coincidentally with the emergence of lethal ranavirosis later in 2011. Whilst we cannot say exactly when *Bd* entered the Serra da Estrela, we are confident that lethal chytridiomycosis emerged at least two years before ranavirosis impacted on the system.

Mortality events attributable to chytridiomycosis in the absence of ranavirosis in the park involved only recently metamorphosed *A. obstetricans*, although other species were found infected and carcasses of two adult *Pelophylax perezi* collected in 2010 were heavily infected with *Bd*^24^. Age-specific mortality of *A. obstetricans* is consistent with findings across Iberia^23^,^37^ and the narrow range of host impacts by *Bd* on amphibians at Serra da Estrela fits with the findings of a recent risk assessment of European amphibians. In that study *Alytes* species were consistently ranked at high risk of infection, while green frogs tended to exhibit prevalence equivalent to background levels^40^.

*Batrachochytrium dendrobatidis* and ranaviruses are known to co-occur and share hosts in amphibian assemblages but attempts to disentangle their interaction and impacts on multi-host communities are not well-described in the literature (refs 46 and 47, but see ref. 32). The negative impacts of lethal ranavirosis we observed in Serra da Estrela were consistent with observations at other locations in Iberia in terms of host range, rapidity of host declines and pathogen genotype (Table S3, Figs 3, 4, 5, 6,^13^,^48^). The phylogenetic relationships of Iberian CMTV-like viruses also appear to reflect geographic patterns; PNTRV’s closest relative is BNV from the region of Spain bordering Portugal. In any case, where these patterns manifested, we could find little evidence that the pre-existing *Bd* infection had an influence on the presence and prevalence of ranavirus infection and the emergence of ranavirosis. Presence of infection with *Bd* and lethal chytridiomycosis was associated with a variety of patterns of ranavirus infection and disease: high levels of co-infection were associated with significant mortality in *Alytes* metamorphs at Repressa da Torre; both pathogens also exhibited low prevalence in association with very low levels of mortality in *Alytes* at Tanque de Sazes; *Bd* circulated at low prevalence but mortality was only associated with ranavirus infection and affected all species present at Tanque de Folgosinho.

It is the observations at Sazes that shift the perspective on ranavirosis in Iberia. Previous to this, Iberian outbreaks of CMTV-like ranaviruses have shown a close association between infection, mortality and multi-host decline suggestive of rapid range expansion and pathogen amplification through a host community after pathogen invasion (Fig. 5^13^). While we have described apparent rapid range expansion in the park (Fig. 2) and observed amplification after invasion associated with mortality events (e.g. Folgosinho and Represa da Torre), infrequent CMTV-like virus infections have been circulating at Tanque de Sazes since at least 2012 without extensive amplification within the community and no evidence of lethal disease (Fig. 5; Table S3). Again, we could find no clear support for an effect of *Bd* on differences in amplification in the host community and broad patterns of infection and disease. For example, the species hardest hit by ranavirosis at Folgosinho (Bosca’s newt) was rarely and only weakly infected with *Bd* (Table 1, S1, Fig. 5) and prevalence of *Bd* did not differ between Folgosinho and Sazes in this species (Fig. 2). We cannot exclude the hypothesis of altitude playing a role on the different patterns found between these two sites (Sazes is <100 m below Folgosinho). This variable has been shown to be a limiting factor in *Bd* host-pathogen systems^23^,^24^.

Although we could not discern any clear pattern of interaction between pre-existing infections with *Bd* and the emergence of CMTV-like ranaviruses in Serra da Estrela Natural Park, their cumulative impacts threaten amphibian communities in the park. Whereas previously the impacts of lethal chytridiomycosis were species- and age-specific, mortality now impacts hosts across all aquatic life history stages (Table S3; Fig. S1) and can drive host communities into precipitous declines (Table 1; Fig. 5). Lethal ranavirosis has increased the number of species threatened with disease-driven declines by at least a factor of four. Greer *et al*.^49^ suggested that the extinction of tiger salamanders as a result of virulent *Ambystoma tigrinum virus* (ATV) was unlikely, with larval salamander populations decreasing and then recovering after ATV-driven epidemics. However, even if PNTRV cannot drive hosts to extinction in Serra de Estrela, we have shown that it severely reduced population sizes to the point where hosts became highly vulnerable to stochastic events^50^: one month after the pond of Folgosinho was cleaned in spring 2014, we only found five adult Bosca’s newts during the breeding season (compared to 228 in 2011) and no overwintered *Alytes* larvae (compared to 126 in 2011).

Although sharp population declines have been observed in all CMTV-like ranavirus outbreaks in Iberia in recent years, host heterogeneity may play an important role in disease dynamics. While ATV can affect ambystomids in North America (e.g. refs 21 and 51) and a similar outcome has been observed in the UK where FV3-like viruses have caused *Rana temporaria* declines^22^, in Iberia we observed entire amphibian assemblages crashing (e.g. ref. 13; this study). These emerging events are taking place in communities that include multiple species from different ectothermic vertebrate classes^13^. Brenes *et al*.^52^ showed experimentally that reptiles and fish live with subclinical infections and therefore might serve as reservoirs for ranaviruses. Equally, non-lethal infections have been documented in lizards (*Iberolacerta monticola*) in Serra da Estrela^53^. Although the virus strain (Lacerta monticolaranavirus, LMRV) detected in lizards is genetically differentiated from the virus we described and has yet to be detected in amphibians in the park, the role of these lizards in PNTRV persistence, plus emergence of a new strain is unclear. However, transmission is possible between different species and vertebrate classes^54^. Emerging hyper-virulent *Ranavirus* strains (e.g. CMTV-like) might in this way take advantage of naïve hosts easing spill-over and species jumps - for example, marbled newts have been reported to prey on Bosca’s newts in our system^55^. This poses an additional threat to all lower vertebrates associated with aquatic habitats, including endemic freshwater fish only found in specific sites in Iberia^56^.

Significant efforts are underway to develop methods to mitigate chytridiomycosis in European amphibians (e.g. ref. 57), but we know of no successful strategy to manage ranavirus infections in captive or wild amphibian populations. CMTV-like ranaviruses and lethal ranavirosis rapidly expand locally (ref. 13; this study) and are extending their reach across Iberia (Fig. 5), home to much of Europe’s amphibian biodiversity, including several endemic species^58^. Unlike other areas on the peninsula affected by CMTV-like ranaviruses, Serra da Estrela Natural Park may hold the key for developing mitigation against this pathogen group. We are the first to describe diverse amphibian communities in Iberia with low-prevalence, circulating CMTV-like virus infections and our hope is that these provide information that can be used to develop real-world solutions for combating amphibian declines caused by ranavirosis.

## Methods

### Study sites and survey design

Serra da Estrela is the highest mountain in the Portuguese mainland territory (maximum altitude 1993 m). It is an extension of the Iberian Sistema Central, located in the eastern part of north-central Portugal^59^,^60^, and comprises the largest protected area in Portugal, Serra da Estrela Natural Park (PNSE; Fig. 2). We surveyed for *Bd* and ranavirus infections in all amphibian species found at 10 locations predominantly located within the park, starting in 2010 and focusing on locations where amphibian mortality events were observed. Specifically, in 2010 we opportunistically sampled live and dead amphibians once in sites where *Bd* had been detected (Table S3; ref. 24). From 2011 onwards we focused our structured field study on two mid-elevation sites with similar geo-climatic features. The first, Folgosinho, is a 255 m^2^ tank located 1079 m a.s.l. where, in 2011, we observed an amphibian mass mortality event. For comparison, we sampled Sazes, a 50 m^2^ tank at 985 m a.s.l. with similar habitat features but where we never observed mass mortality events. Both are constantly fed with spring water, and both are approximately 1.5 meters deep. We sampled the two focal sites three to four times per year (once in spring, summer, autumn and, depending on the weather, also winter) for two to three days (each time) using a standardized effort (2 persons/2 hours/day/site). We sampled a maximum of 3 meters from the pond margin using 50 cm dip nets. Other areas of the natural park were also surveyed opportunistically, dip netting between 1 and 2 hours (Fig. 2; Table S3; see ref. 24).

We recorded visible signs of disease for both ranavirosis (see e.g. refs 13 and 26) and chytridiomycosis^61^. All live specimens were skin swabbed for *Bd* screening^62^,^63^ and we collected a small piece of tail tissue or toe clip, which was stored in 70% ethanol for ranavirus screening - using PCR^64^ – and skeletochronology (Further details are provided in Supplementary information, *SI Appendix*). Before release, we applied antiseptic and pain relieving solution (Bactine^®^, Bayer, USA) to the clipped toes as an analgesic and disinfectant^65^. We took liver and skin tissue samples from corpses and stored them in 90% ethanol for respective molecular detection of ranavirus and *Bd*. A selected number of carcasses collected during the first outbreak were stored in 70% ethanol for post-mortem analyses.

Water quality analyses were not indicative of environmental contamination^66^. To reduce the risk of spreading pathogens across sites, we used disposable vinyl gloves to handle animals and disinfected field equipment and hiking boots in a 1% solution of Virkon^®^ (Antec International ltd., Sudbury, Suffolk, UK) between sites^67^.

### Disease screening, *Ranavirus* sequencing and phylogenetics

Dead Bosca’s newts were necropsied, although examination was impaired by the advanced autolysis of some animals. Histological examination of tissue samples was attempted after fixation in 10% phosphate-buffered formalin and embedded in paraffin.

We extracted DNA from tissue samples (skin and liver) using the DNeasy Blood & Tissue Kit (Qiagen, Hilden, Germany). Swabs and skin extractions were screened for the presence of *Bd* using quantitative real-time polymerase chain reaction (qPCR), following the protocol of Boyle *et al*.^68^. PCR to detect *Ranavirus* was performed on the DNA samples using the MCP4 and 5 primers targeting the viral MCP gene (CMTV ORF 16L; major capsid protein; AFA44920) as described by Mao *et al*.^69^. Samples that tested positive for *Ranavirus* were subjected to additional PCR reactions to amplify partial sequences from CMTV ORFs 22L (GenBank accession number AFA44926), 58L (AFA44964), 59R (AFA44965), 82L (AFA44988), and a region covering a noncoding sequence and the start of 13R (AFA44917) (see Table S1). PCR amplicons were submitted to Beckman Coulter Genomics for Sanger sequencing of both DNA strands. Additional sequences were downloaded from GenBank (see Table S2).

We visually confirmed base calls by examining electropherograms in CodonCode Aligner (http://www.codoncode.com/aligner/). Forward sequences were reverse complemented and aligned to reverse sequences using PRANK v.100802^70^. The aligned forward and reverse sequences for each sample were then viewed in Jalview 2.8^71^ and ambiguous base calls were corrected with reference to the electrophoretograms of both sequences. Sequences were aligned to published *Ranavirus* sequences downloaded from the NCBI nucleotide database, again using PRANK with default settings. All gaps were removed from the alignments with trimAl^72^ prior to concatenation with PhyUtility^73^. Trees were constructed from a partitioned alignment using both MrBayes 3.2.2^74^ and RAxML^75^ with the GTR model of nucleotide substitution and rate variation among sites modelled by a discrete gamma distribution with four categories. We ran MrBayes for 750000 generations with default settings (4 chains, 2 runs, sample frequency = 500, and a 25% burn-in). Twenty maximum-likelihood trees were generated on distinct starting trees in RAxML. Node support values (posterior probabilities [Mr. Bayes] and 100 bootstraps [RAxML]) were annotated on the best maximum-likelihood tree.

### Statistical analysis

We selected *L. boscai* and *A. obstetricans* (the two most abundant species) to assess variation in the prevalence of *Bd* over time per site and within sites using a general linear model (GLM), with prevalence of ranavirus as covariate (JMP PRO 12.0; SAS Institute Inc). Accounting for the ranavirus emergence and prevalence allowed understanding the contribution of this second pathogen to the variation on *Bd* prevalence.

Density was calculated using maximum abundance on a single day per life stage per sampling season and dividing it by the area of the aquatic habitat (highest *n*/area). Time series of counts were analysed for overall trends in population size using Poisson regression (log-linear models^76^) with the software TRIM3.0^77^. We used the linear trend model with all years as change points, except for years with no observations. We plotted overall trend estimates for *A. obstetricans* larvae, *L. boscai* adults, *S. salamandra* larvae and *T. marmoratus* adults, calculated as the slope of the regression line through the logarithms of the indices over the whole study period. We used 95% confidence intervals of the overall trend estimate to test for significant population trends for each species (=slope + /−1.96 times the standard error of the slope^78^). We followed trend classification proposed by van Strien *et al*.^44^ where (e.g.) “*substantial decline/steep decline”* represents a decline significantly more than 5% per year (5% would mean a halving in abundance within 15 years), and “*uncertain”* is no evidence of a significant increase or decline in the population, or if trends are less than 5% per year.

## Additional Information

**How to cite this article:** Rosa, G.M. *et al*. Impact of asynchronous emergence of two lethal pathogens on amphibian assemblages. *Sci. Rep.* **7**, 43260; doi: 10.1038/srep43260 (2017).

**Publisher's note:** Springer Nature remains neutral with regard to jurisdictional claims in published maps and institutional affiliations.

## Supplementary Material

Supplementary Information

## Figures and Tables

**Table 1:** Epidemiology and demographic trends of two amphibian assemblages at Serra da Estrela (Portugal) after ranavirosis outbreaks.

Site	Epidemiology			Population monitoring		
	Ranavirosis infection	Mortality	Species	Life stage	Slope (SE)	Population trend
Folgosinho	yes (all species)	yes	*A. obstetricans*	overwintering larvae	−0.3012 (0.0816)	Substantial decline (p < 0.01)**
			*L. boscai*	adults	−0.9883 (0.1388)	Substantial decline (p < 0.01)**
			*T. marmoratus*	adults	−0.5562 (0.2296)	Substantial decline (p < 0.01)**
			*S. salamandra*	larvae	0.0375 (0.4007)	Uncertain
Sazes	yes (only on *S. salamandra* and *L. boscai*)	no	*A. obstetricans*	overwintering larvae	0.2045 (0.1174)	Uncertain
			*L. boscai*	adults	−0.0094 (0.0800)	Uncertain
			*T. marmoratus*	adults	0.1590 (0.0956)	Uncertain
			*S. salamandra*	larvae	−0.0373 (0.1376)	Uncertain

Trend classification follows TRIM v.3.53 (see also ref. 44) where the multiplicative overall slope is converted into a category. The category depends on the slope as well as its 95% confidence interval.
