# Supplementary material for: Impact of asynchronous emergence of two lethal pathogens on amphibian assemblages
Source: Sci Rep. 2017 Feb 27;7:43260. doi: 10.1038/srep43260 (PMC5327436; doi:10.1038/srep43260)
Supplement: Supplementary Information [file srep43260-s1.pdf]

SUPPLEMENTARY INFORMATION

OPEN

SCIENTIFIC REPORTS

| 7:43260 | DOI: 10.1038/srep43260

Impact of asynchronous emergence of two lethal pathogens on amphibian assemblages

Gonçalo M. Rosa, Joana Sabino-Pinto, Telma G. Laurentino, An Martel, Frank Pasmans, Rui Rebelo, RichardA. Griffiths, Anke C. Stöhr, Rachel E. Marschang, Stephen J. Price, Trenton W. J. Garner & Jaime Bosch

Methods

PCR detection of viral agent. PCR to detect *Ranavirus* was performed on the DNA samples using the MCP4 and MCP5 primers targeting the viral MCP gene. Samples that tested positive for *Ranavirus* were subjected to additional PCR reactions to amplify partial sequences. Primers provided in the table S1.

Target gene	Locus (CMTV ORF ref.)	Nucleotide sequence (5' to 3')	
		forward primer	reverse primer
Hypothetical protein gene	13R	CTTCCCGTGTCTGGGTTGA	TGCACTCCGTAGCTCCTAAG
Proliferating cell nuclear antigen gene	22L	CAGTCCGTGTCTGTCGTAGA	CTCCGAAAACACCCAGGTTC
p31k gene	82L	ATCCTCTTTTCTTTCGGCGC	CCCTGCACTTTTCCTTGACC
Hypothetical protein gene	58L	CCATGTACCCTCAGACCCTG	CATAGTCCGAACCCAAAGCG
Hypothetical protein gene	59R	GCATAGAGACGGATACAAGCG	GAAACAAGGCCGCTCTAGTC
Major capsid protein gene (69)	16L	GTCTCTGGAGAAGAAGAA	GACTTGGCCACTTATGAC

Table S1. Primers used for the successful amplification of *Ranavirus* DNA from the infected tissues.

***Ranavirus* phylogenetics.** Additional sequences from previously characterized ranaviruses were downloaded from GenBank: *Ambystoma tigrinum virus* (ATV, GenBank accession number AY150217), Ándaran Alytes obstetricans virus (AAOV, KJ703123), Andrias davidianus ranavirus (ADRV, KC865735.1), Bosca's newt virus (BNV, KJ703122), Common midwife toad virus (CMTV, JQ231222), Common midwife toad virus (Netherlands) (CMTV (nl), KP056312), *Epizootic hematopoietic necrosis virus* (EHNV, FJ433873), European sheatfish virus (ESV, JQ724856), *Frog virus 3* (FV3, AY548484), German gecko ranavirus (GGRV, KP266742), Rana grylio virus (RGV, JQ654586), Soft-shelled turtle iridovirus (STIV, NC012637), Spotted salamander Maine (SsME, KJ1751441), Testudo hermanni ranavirus (THRV - previously CH8/96 -, KP266741), Tiger frog virus (TFV, AF389451), and Tortoise ranavirus 1 (ToRV1, KP266743). GenBank accession numbers of new ranavirus sequences obtained during this study are provided in the table S2.

Sample ID	Site	Year	Host	Accession numbers by locus (CMTV ORF ref.)					
				13R	16L	22L	58L	59R	82L
G20	LCQ	2013	*S. salamandra*	KY207392	KY207437	KY207401	KY207419	KY207428	KY207410
G23	LCQ	2013	*L. boscai*	KY207393	KY207438	KY207402	KY207420	KY207429	KY207411
G6	TGS	2012	*A. obstetricans*	KY207394	KY207439	KY207403	KY207421	KY207430	KY207412
H61	TGS	2013	*T. marmoratus*	KY207395	KY207440	KY207404	KY207422	KY207431	KY207413
H71	TGS	2013	*S. salamandra*	KY207396	KY207441	KY207405	KY207423	KY207432	KY207414
I23	LCS	2013	*L. boscai*	KY207397	KY207442	KY207406	KY207424	KY207433	KY207415
I55	RTR	2013	*B. spinosus*	KY207398	KY207443	KY207407	KY207425	KY207434	KY207416
J14	RTR	2011	*A. obstetricans*	KY207399	KY207444	KY207408	KY207426	KY207435	KY207417
LMRV	PCL	2003 / 2004	*I. monticola*	KY207400	KY207445	KY207409	KY207427	KY207436	KY207418

Table S2. GenBank accession numbers of amphibian ranaviruses obtained during this study at Serra da Estrela, Portugal. References for loci relate to CMTV complete genome (JQ231222). Abbreviations key: LCQ, Lagoa do Covão das Quelhas; LCS, Lagoa dos Cântaros; PCL, Planalto Central; RTR, Represa da Torre; FGS, Tanque de Folgosinho.

Newt skeletochronology. Newt specimens were sexed and measured from the tip of the snout to the posterior margin of the cloaca (snout-vent length: SVL) to the nearest 0.5 mm. Mis-assignment of sex of a few specimens in the field was corrected during exploratory data analysis. The right humerus and a phalanx of toe 4 of the right hind-limb were removed for skeletochronology purposes and also stored in 70% ethanol. Although the exact count of LAG is more difficult in phalanges than in humeri, it is possible to age newts through the analysis of the phalanges[79]. Thus, for ethical reasons, humeri were used just to assess age of dead specimens, while a phalanx of toe 4 (right hind-limb) was collected from live specimens. This meant that no live animals had to be sacrificed and minimized any possible increase in susceptibility to infections or predation. The use of skeletochronology allowed detection of any age or life stage specific patterns in mortality. Humeri and phalanges were decalcified in 3% nitric acid for 10 min (phalanx) and 50 min (humerus), cross-sectioned (14 µm width) and stained with Ehrlich's haematoxylin for 20 min (more details in ref. 80, 81). The sections were obtained after mounting on Sakura Tissue-Tek® O.C.T Compound, on a Clinicut 60 cryostat. The bone sections were fixed in a microscope slide with and posteriorly photographed and analysed.

Lines of arrested growth (LAGs) present in the periosteal bone were considered to correspond to periods of inactivity, and the zones of bone layers between LAG correspond to the periods of activity and growth[79,82]. A non-periodic line of metamorphosis has never been observed for this species in Portugal[79,83]. Therefore, age can be estimated by directly counting the LAGs in the periosteal bone[80]. The presence of additional lines, which could have been reabsorbed by the growth of the endosteal bone and the advancing cementing resorption line, was determined by measuring the average diameter of the first year LAG in the young individuals.

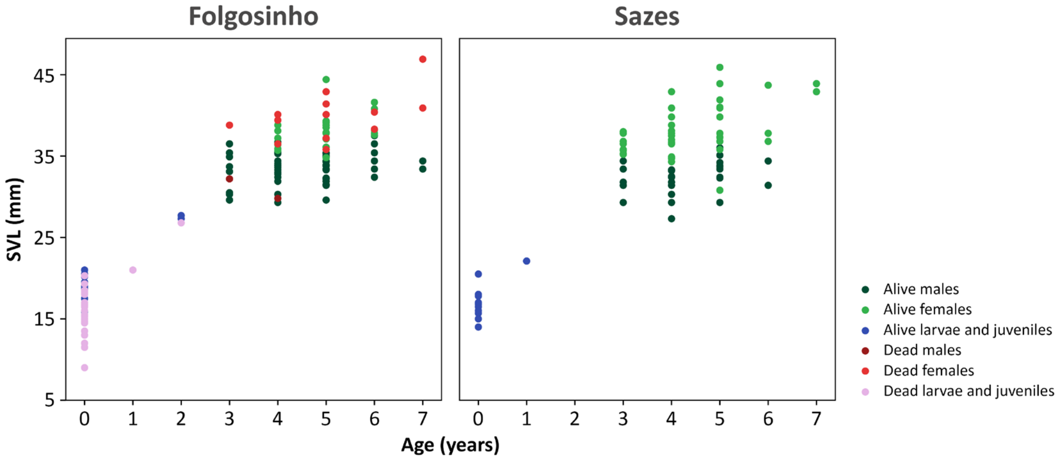

Figure S1. The relationships between age and size (snout-vent length, SVL) of *L. boscai* plotted by population, between 2011 and 2014: Folgosinho illustrates the population of newts where yearly outbreaks of ranaviruses have occurred, affecting all ages (reddish tones highlight individuals found dead and positive for the pathogen), while Sazes is used to illustrate a comparative population where outbreaks have not been recorded. Mortality in Folgosinho was found on both males and females.

Results

Newt skeletochronology. The skeletochronological analysis of the *L. boscai* populations showed that the same number of LAG in humeri and phalanges were confirmed for almost all the individuals where both bones were analysed; however in eight individuals (out of 210) the phalanges exhibited one LAG less, which is expected due to a natural higher rate of endosteal resorption in phalanges[82]. Similar results have been previously shown also for *L. boscai*[84]. Larvae and recently metamorphosed individuals that were caught before the first season of low activity showed no LAGs. The results from this analysis showed that mortality occurred across all life stages and ages within stages making use of the aquatic environment at Folgosinho, from larval forms to recent metamorphs or sexually mature adults (Fig. S1).

References

79. Caetano MH, Castanet J. Variability and microevolutionary patterns in *Triturus marmoratus* from Portugal: age, size, longevity and individual growth. *Amphib-Reptilia* **14,** 117–129 (1993).
80. Castanet J, Smirina E. Introduction to the skeletochronological method in amphibians and reptiles. *Ann. Sci. Nat. Zool.* **13e,** Series 11, 191–196 (1990).
81. Rebelo R, Caetano MH. Use of the skeletochronological method for ecodemographical studies on *Salamandra salamandra gallaica* from Portugal. *Scientia Herpetologica* (eds. Llorente GA, Montori A, Santos X, Carretero MA), 135–140 (1995).
82. Caetano MH. Use and results of skeletrochronology in some urodeles (*Triturus marmoratus*, Latreille 1800 and *Triturus boscai*, Lataste 1879). *Ann. Sci. Nat. Zool.* **13e,** Series 11, 197–199 (1990).
83. Caetano MH, Leclaire R. Comparative phenology and demography of *Triturus boscai* from Portugal. *J. Herpetol.* **33,** 192–202 (1999).
84. Díaz-Paniagua C, Mateo JA. Geographic variation in body size and life history traits in *Triturus boscai*. *Herpetol. J.* **9,** 21–27 (1999).

Site	Geographic coordinates	Year	Host species	Life stage	Ranavirus		Batrachochytrium dendrobatidis	
					prevalence	95% CI (range)	prevalence	95% CI (range)
Charco da Pedreira de Santa Comba de Seia	40°27'30.22"N, 7°42'36.41"W; 475 m a.s.l.	2013	Pp	L	0/3 (0)	0.000-0.561		
				A	0/4 (0)	0.000-0.490		
			Pc	L	0/2 (0)	0.000-0.658		
Erva da Fome	40°23'28.87"N, 7°36'1.00"W; 1450 m a.s.l.	2014	Ri	L	0/5 (0)	0.000-0.435	0/5 (0)	0.000-0.435
			Ss	L	1/1 (100)	0.207-1.000	0/1 (0)	0.000-0.794
Lagoa dos Cântaros	40°20'9.43"N, 7°35'33.20"W; 1646 m a.s.l.	2010	Ao	L			17/19 (89.5)	0.686-0.971
				M	0/1 (0)	0.000-0.794	1/1 (100)	0.207-1.000
		2011	Ao	L			16/16 (100)	0.806-1.000
			Pp	L			0/5 (0)	0.000-0.435
				A			0/3 (0)	0.000-0.562
		2012	Ao	L	0/1 (0)	0.000-0.794	0/1 (0)	0.000-0.794
			Pp	L	1/1 (100)	0.207-1.000		
			Tm	L	0/3 (0)	0.000-0.562		
		2013	Pp	L	0/15 (0)	0.000-0.204	0/4 (0)	0.000-0.490
				J	0/4 (0)	0.000-0.490		
				A	0/3 (0)	0.000-0.562		
			Tm	L	0/6 (0)	0.000-0.390		
			Lb	L	1/1 (100)	0.207-1.000		
Lagoa do Covão das Quelhas	40°19'38.55"N, 7°37'31.81"W; 1820 m a.s.l.	2011	Ao	A			1/1 (100)	0.207-1.000
			Bs	A			1/2 (50)	0.095-0.906
			Pp	L			0/5 (0)	0.000-0.435
				A			0/7 (0)	0.000-0.354
			Hm	A			1/2 (50)	0.095-0.906
			Ss	M			0/4 (0)	0.000-0.490
				A			0/2 (0)	0.000-0.658
			Tm	J			1/2 (50)	0.095-0.906
			Lb	A			0/2 (0)	0.000-0.658
		2012	Ao	M			1/5 (20)	0.036-0.625
		2013	Ao	M	0/2 (0)	0.000-0.658	0/3 (0)	0.000-0.562
			Pp	L	0/8 (0)	0.000-0.324		
				J	0/2 (0)	0.000-0.658		
			Ss	L	1/1 (100)	0.207-1.000	0/1 (0)	0.000-0.794
				J	0/2 (0)	0.000-0.658	0/2 (0)	0.000-0.658
			Tm	A	0/1 (0)	0.000-0.794		
			Lb	M	0/1 (0)	0.000-0.794		
				A	2/2 (100)	0.342-1.000	0/1 (0)	0.000-0.794
Represa da Torre	40°19'34.61"N, 7°36'32.07"W; 1955 m a.s.l.	2010	Ao	M	0/4 (0)	0.000-0.490	4/13 (31)	0.127-0.576
		2011	Ao	L			22/23 (96)	0.790-0.992
				M	4/4 (100)	0.510-1.000	20/21 (95)	0.773-0.992
			Bs	M	9/9 (100)	0.701-1.000	1/9 (11)	0.020-0.435
			Pp	L			3/3 (100)	0.439-1.000
				A			0/1 (0)	0.000-0.794

Table S3. Summary of samples screened for *Ranavirus* and *Batrachochytrium dendrobatidis* in Serra da Estrela (Portugal) by site and year (2011-2014). Prevalence includes 95% confidence intervals (CIs). Life stages: L, larvae; M, metamorphs; J, juveniles; A, adults Species: Ao, *Alytes obstetricans*; Bs, *Bufo spinosus*; Pc, *Pelobates cultripes*; Pp, *Pelophylax perezi*; Ri, *Rana iberica*; Hm, *Hyla molleri*; Ss, *Salamandra salamandra*; Tm, *Triturus marmoratus*; Lb, *Lissotriton boscai*.

		2012	Ao	L			1/1 (100)	0.207-1.000
				M	0/1 (0)	0.000-0.794	1/1 (100)	0.207-1.000
			Bs	M	4/24 (16.7)	0.067-0.359	22/24 (92)	0.742-0.977
			Pp	L			6/7 (86)	0.487-0.974
				M	0/2 (0)	0.000-0.658	2/3 (67)	0.207-0.939
				A			1/2 (50)	0.095-0.906
		2013	Ao	L	16/22 (72.7)	0.518-0.868	11/21 (52)	0.324-0.717
				M	1/5 (20)	0.036-0.624	5/6 (83)	0.437-0970
			Bs	M	1/1 (100)	0.207-1.000	1/1 (100)	0.207-1.000
			Pp	L	0/5 (0)	0.000-0.435	2/5 (40)	0.118-0.769
				A	0/4 (0)	0.000-0.490	1/4 (25)	0.046-0.699
		2014	Pp	A			0/1 (0)	0.000-0.794
Represa de Sazes	40°20’39.14”N, 7°43’21.78”W; 780 m a.s.l.	2010	Ao	L			3/20 (15)	0.052-0.360
		2012	Ao	L			0/26 (0)	0.000-0.129
			Lb	A	0/1 (0)	0.000-0.794		
		2013	Ao	L	0/17 (0)	0.000-0.184		
			Ri	L	0/5 (0)	0.000-0.435		
				A	0/3 (0)	0.000-0.562		
			Ss	L	0/6 (0)	0.000-0.390	0/6 (0)	0.000-0.390
				M	0/1 (0)	0.000-0.794	0/1 (0)	0.000-0.794
			Lb	A	0/1 (0)	0.000-0.794		
Salgadeiras	40°20'18.84"N, 7°36'51.59"W; 1845 m a.s.l.	2010	Pp	A	0/1 (0)	0.000-0.794	0/1 (0)	0.000-0.794
		2014	Ao	A	1/14 (7.1)	0.013-0.315	4/14 (29)	0.117-0.547
			Hm	A	1/1 (1)	0.207-1.000	1/1 (100)	0.207-1.000
			Pp	A	1/12 (0)	0.015-0.354	1/12 (0)	0.015-0.354
			Ss	A	0/1 (0)	0.000-0.794	0/1 (0)	0.000-0.794
Tanque do Alvoco	40°17’59.37”N, 7°41’21.32”W; 861 m a.s.l.	2010	Ao	L			1/3 (33.3)	0.062-0.792
		2012	Ao	L			17/25 (68)	0.484-0-828
			Tm	J			0/2 (0)	0.000-0.658
			Lb	A	0/1 (0)	0.000-0.794	1/4 (25)	0.046-0.699
		2013	Lb	A	1/1 (100)	0.207-1.000	0/1 (0)	0.000-0.794
Tanque de Folgosinho	40°29’37.09”N, 7°31’47.61”W; 1079 m a.s.l.	2010	Ao	L			7/19 (36.8)	0.000-0.658
				A	0/2 (0)	0.000-0.658	0/2 (0)	0.000-0.658
		2011	Ao	L	2/2 (100)	0.342-1.000	3/52 (5.8)	0.020-0.156
				M	1/1 (100)	0.207-1.000	0/4 (0)	0.000-0.490
				A	0/2 (0)	0.000-0.658	4/15 (26.7)	0.109-0.520
			Tm	A	2/2 (100)	0.342-1.000	0/4 (0)	0.000-0.490
			Lb	L	20/20 (100)	0.839-1.000	0/2 (0)	0.000-0.658
				M	10/10 (100)	0.723-1.000	1/8 (12.5)	0.022-0.471
			Ss	A	40/80 (50.0)	0.393-0.607	1/24 (4.2)	0.007-0.202
				L	0/6 (0)	0.000-0.390		
				M			0/1 (0)	0.000-0.794
		2012	Ao	L	2/3 (66.7)	0.208-0.939	2/9 (22)	0.063-0.547
				A	3/3 (100)	0.439-1.000		
			Tm	L	1/1 (100)	0.207-1.000	0/1 (0)	0.000-0.794
				A	1/3 (33.3)	0.061-0.792	0/8 (0)	0.000-0.324
			Lb	L	17/18 (94.4)	0.742-0.990		
				M	4/15 (26.7)	0.109-0.520		
				A	26/104 (25.0)	0.177-0.341	2/43 (5)	0.013-0.155

Table S3. Continued.

			Ss	L	0/10 (0)	0.000-0.278	0/9 (0)	0.000-0.168
				A	0/1 (0)	0.000-0.794	0/1 (0)	0.000-0.794
		2013	Ao	L	4/9 (44.4)	0.189-0.733	0/9 (0)	0.000-0.168
				M	0/1 (0)	0.000-0.794	0/1 (0)	0.000-0.794
				A	0/5 (0)	0.000-0.435	0/5 (0)	0.000-0.435
			Tm	L	0/1 (0)	0.000-0.794	0/1 (0)	0.000-0.794
				A	1/18 (5.6)	0.010-0.258		
			Lb	L	5/9 (55.6)	0.267-0.811	0/4 (0)	0.000-0.490
				M	3/7 (42.9)	0.158-0.750	0/4 (0)	0.000-0.490
				A	10/76 (13.2)	0.073-0.226	0/2 (0)	0.000-0.658
			Ss	L	2/19 (10.5)	0.029-0.314	0/19 (0)	0.000-0.168
				M	0/2 (0)	0.000-0.658	0/2 (0)	0.000-0.658
		2014	Ao	L	0/6 (0)	0.000-0.390	2/6 (33)	0.097-0.700
				A	0/6 (0)	0.000-0.390	0/6 (0)	0.000-0.390
			Tm	A	0/2 (0)	0.000-0.658	2/2 (100)	0.342-1.000
			Lb	A	0/5 (0)	0.000-0.435	0/5 (0)	0.000-0.435
Tanque dos Serviços Florestais de Sazes	40°20’39.70”N, 7°42’52.63”W; 985 m a.s.l.	2010	Ao	L			12/20 (60)	0.387-0.781
		2011	Ao	L	0/4 (0)	0.000-0.490	2/34 (6)	0.016-0.191
			Lb	L	0/12 (0)	0.000-0.243		
				A	0/25 (0)	0.000-0.133	2/24 (8)	0.023-0.259
			Tm	A			0/7 (0)	0.000-0.354
			Ss	M			0/1 (0)	0.000-0.794
		2012	Ao	L			1/28 (4)	0.006-0.177
			Lb	L	0/36 (0)	0.000-0.096		
				A	0/45 (0)	0.000-0.079	0/24 (0)	0.000-0.138
				L	0/2 (0)	0.000-0.658	0/1 (0)	0.000-0.794
			Tm	J			0/1 (0)	0.000-0.794
			Ss	A	0/6 (0)	0.000-0.390	0/18 (0)	0.000-0.176
				L	0/3 (0)	0.000-0.561	0/3 (0)	0.000-0.562
				A	2/5 (40)	0.118-0.769	0/4 (0)	0.000-0.490
		2013	Ao	L	0/35 (0)	0.000-0.099	6/42 (14)	0.067-0.278
			Ri	A	0/1 (0)	0.000-0.794		
			Lb	L	0/21 (0)	0.000-0.155		
				M	0/2 (0)	0.000-0.658		
				A	0/80 (0)	0.000-0.046	0/3 (0)	0.000-0.562
			Tm	L	0/13 (0)	0.000-0.228	0/4 (0)	0.000-0.490
				A	0/23 (0)	0.000-0.143		
			Ss	L	0/33 (0)	0.000-0.104	0/33 (0)	0.000-0.104
				A	0/1 (0)	0.000-0.794	0/1 (0)	0.000-0.794
		2014	Ao	L	0/12 (0)	0.000-0.243	0/15 (0)	0.000-0.204
			Lb	A	2/12 (16.7)	0.047-0.448	0/12 (0)	0.000-0.243
			Tm	A	0/10 (0)	0.000-0.278	0/10 (0)	0.000-0.278
			Ss	L	0/3 (0)	0.000-0.561	0/3 (0)	0.000-0.561

Table S3. Continued.
